# Non-Uniform Microstrip Antenna Array for DSRC in Single-Lane Structures

**DOI:** 10.3390/s16122101

**Published:** 2016-12-11

**Authors:** Tiago Varum, João N. Matos, Pedro Pinho

**Affiliations:** 1 Instituto de Telecomunicações, Campus Universitário de Santiago, 3810-135 Aveiro, Portugal; matos@ua.pt (J.N.M.); ppinho@deetc.isel.pt (P.P.); 2 Universidade de Aveiro, Campus Universitário de Santiago, 3810-135 Aveiro, Portugal; 3 Instituto Superior de Engenharia de Lisboa, Rua Conselheiro Emílio Navarro, 1959-009 Lisboa, Portugal

**Keywords:** dedicated short range communications, antenna arrays, microstrip antennas

## Abstract

Vehicular communications have been subject to a great development in recent years, with multiple applications, such as electronic payments, improving the convenience and comfort of drivers. Its communication network is supported by dedicated short range communications (DSRC), a system composed of onboard units (OBU) and roadside units (RSU). A recently conceived different set-up for the tolling infrastructures consists of placing them in highway access roads, allowing a number of benefits over common gateway infrastructures, divided into several lanes and using complex systems. This paper presents an antenna array whose characteristics are according to the DSRC standards. Additionally, the array holds an innovative radiation pattern adjusted to the new approach requirements, with an almost uniform wide beamwidth along the road width, negligible side lobes, and operating in a significant bandwidth.

## 1. Introduction

The evolution of wireless communications has been reflected in several areas, including transport networks, wherein the development of vehicular communications has become evident in recent years, offering several functionalities to the drivers and other travelers, allowing the improvement of important safety issues, reducing traffic jams, or even accidents.

Dedicated short range communications (DSRC) is the technology enabling wireless communication among onboard units (OBUs) or between OBUs and roadside units (RSUs), allowing vehicle-to-vehicle and vehicle-to-infrastructure communications, being regulated by a set of standards. Electronic payments for tolls, parking, or gas stations are an application with increasing development in vehicular communications, due to the convenience and comfort that it provides to users.

The electronic toll collection applied to highways is usually made based on input and output road logs, or using several gateways along the highway. These structures cover various lanes and allow quick access and the fluency of traffic. However, they involve at least one system covering each lane, and also require great care to overcome potential interferences between the various RSU’s, or with the vehicles passing in adjacent lanes.

**Figure 1:**
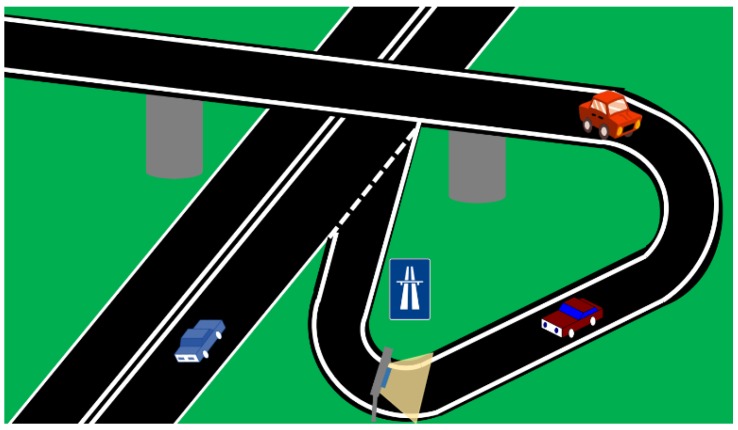
Highway tolling system in access lane.

A recent approach suggests that the RSU—s gateways could be placed in the input and output access roads of the highways (typically single lane) as shown in Figure 1. This concept allows the use of only one RSU system, avoiding problems of interference between lanes and a simpler system, since in these lanes—commonly with curved shape—the speeds are much lower than in the highways.

The CEN EN12253 [1] standard provides a set of constraints to the physical medium of DSRC, including for the RSU antenna. It mentions that the antenna must operate at least in a 20 MHz band around 5.8 GHz, with a maximum equivalent isotropic radiated power of +33 dBm up to an angle of 70° with the vertical (outside this zone may not exceed +18 dBm). This implies antenna sidelobes with a level at least 15 dB below the maximum. In terms of polarization, it should be left hand circular (LHCP) with a rejection of 15 dB in boresight, and 10 dB in the zone where the gain drops 3 dB.

Furthermore, since the access roads tend to be wider than the highway lanes, the specific application imposes a radiation pattern on the road plane (horizontal) larger than the usual DSRC tolls applications, and with a uniform gain across the width to avoid communication failures at the ends or passing without detection.

Some antenna arrays targeted to RSU of DSRC systems are proposed [2,3,4,5,6,7,8]. It is presented a 4 × 4 right hand circularly polarized antenna array, with microstrip elements having an H-shape with a T-slot [2]. This array was designed for 5.8 GHz, showing a bandwidth of 109 MHz, sidelobes below −25 dB, and about 22° beamwidth. However, the design using a multi-layer structure inserts some complexity in the design, in the manufacture, and increases the cost. A 4 × 4 array with square microstrip elements is presented [3]. This array with a 500 × 500 mm^2^ structure size applies the sequential rotation technique to produce circular polarization. In terms of sidelobes, these are higher than desired (between −10 and −15 dB), whereby the authors use an absorbent Korean paper with Chinese ink, after the design to reach values on the order of −20 dB. The complexity of the final structure making it possible to obtain reasonable results concordant with the DSRC standards is the main disadvantage of this solution.

An array is presented with four double loop monopoles for a DSRC system [4]. This antenna has 600 MHz bandwidth, beamwidth of 25°, and sidelobes below −19 dB. However, it has a large ground plane (90 × 90 cm^2^) and the absence of circular polarization. For a free-flow system, a microstrip patch superimposed by a metamaterial layer designed using square split-ring resonator elements, which increase the directivity of the single patch [5]. The authors propose a four-patch configuration to obtain a radiation pattern with a 36° beamwidth and a directivity of 16.38 dBi. The complexity and volume make this structure unattractive. In addition, for the infrastructure of a DSRC system, reference [6] presents a Fabry–Perot resonator antenna consisting of a circularly polarized square patch and a partially reflective superstrate. The main characteristics of the antenna are a gain of 18 dBi, sidelobe level (SLL) of −16 dB, and 21° beamwidth. However, its complex structure and its volume are the main disadvantages. In reference [7], a 2 × 4 microstrip antenna array is presented which combines reduced complexity and size. This array has as its main feature a wide operating bandwidth covering the DSRC bands, in addition to a radiation diagram complying with the standards. However, it has sidelobes that are slightly higher than desired.

Finally, in reference [8], an antenna that overcomes the main problems revealed by the other reported antennas is shown, largely fulfilling the constraints of DSRC standards. Despite its design complexity, this 12 element array has a single-layer structure, which allows for simplicity of manufacture and reduced cost, as well as low weight and volume. In addition, the array presents a wide bandwidth (greater than 1 GHz band) covering the DSRC bands, reduced sidelobes, and a radiation pattern with a beamwidth around 22°. All of the proposed antennas in the literature focus on the common toll gates and free flow configurations, showing radiation patterns with narrow half power beam widths (HPBW) in the horizontal plane, in addition to highly complex structures.

Taking the DSRC and the application requirements into account, this paper presents an innovative array whose characteristics fit the needs for tolling application in access roads of highways, including its wider radiation pattern, a uniform gain across the road width, and with reduced sidelobes. Furthermore, the entire array was designed using microstrip structures, with the advantages of being light weight, simple, and low cost.

This paper is divided into four sections. It begins with an introduction to vehicular communications (focusing on tolling applications), and stating the objectives. The second section describes the design of the proposed antenna array, and Section 3 shows the simulation and measurement results. Finally, the last section reports the main conclusions.

## 2. Antenna Array Design

Due to their benefits (such as their simplicity and versatility), microstrip antennas [9] are frequently used in many wireless applications, achieving low profile antennas with a simple and inexpensive manufacture. Moreover, they enable the design of array feed networks (AFN) using microstrip transmission lines in the plane of the array elements, allowing single-layer structures.

### 2.1. Antenna Array Element

**Figure 2:**
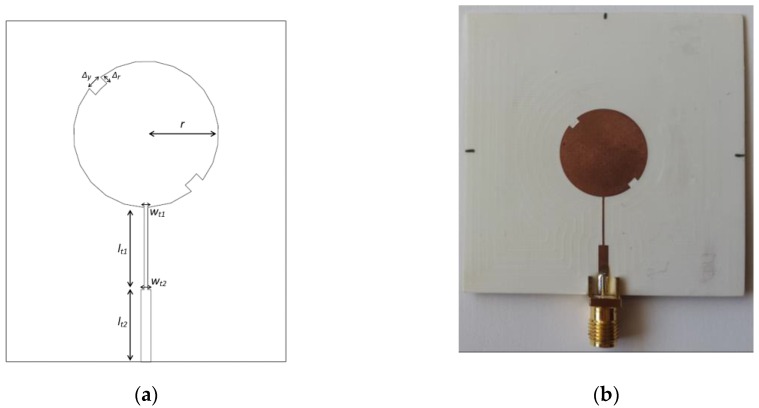
Microstrip patch element: (**a**) structure and dimensions; (**b**) photograph of the prototype.

The antenna element used consists of a circular microstrip patch (as shown in Figure 2), whose resonance frequency varies with the radius *r*. Additionally, two side slits (*Δ_y_ × Δ_r_*) were created for LHCP generation [9]. To adapt the antenna input impedance to the impedance of AFN transmission lines, a double quarter wavelength transformer was inserted (*l_t1_* and *l_t2_*).

The dimensions of the microstrip element designed for 5.8 GHz are: *r* = 9.1 mm, *Δ_y_* = 2 mm, *Δ_r_* = 1.1 mm, *l_t1_* = 10.2 mm, *w_t1_* = 0.45 mm, *l_t2_* = 9 mm, and *w_t2_* = 1.25 mm. The circular patch element was manufactured, and the prototype is shown in Figure 2b. It was measured in terms of its main parameters, and results were compared with those obtained by simulation (presented below).

**Figure 3:**
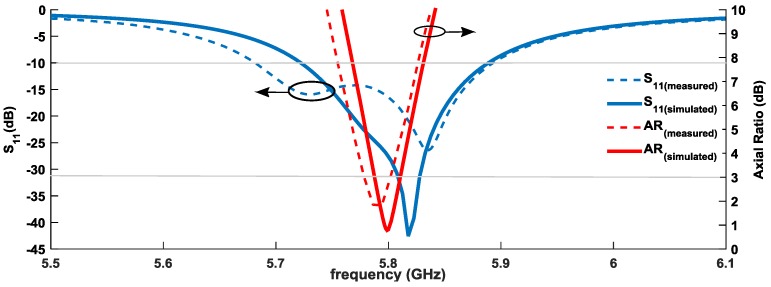
Simulated and measured S_11_ and axial ratio (AR) of the designed circular patch antenna.

Figure 3 shows a comparison between the simulated and measured results, both of the S_11_ and axial radio of the manufactured antenna. It is possible to observe a good agreement between simulated and measured curves. In terms of S_11_—assuming the usual criterion for a good impedance matching an S_11_ < −10 dB—the patch has a measured bandwidth of 210 MHz (5.68–5.89 GHz). Considered as a good circularly polarized an antenna with an axial ratio lower than 3 dB, the circular patch has a measured 50 MHz bandwidth of circular polarization.

**Figure 4:**
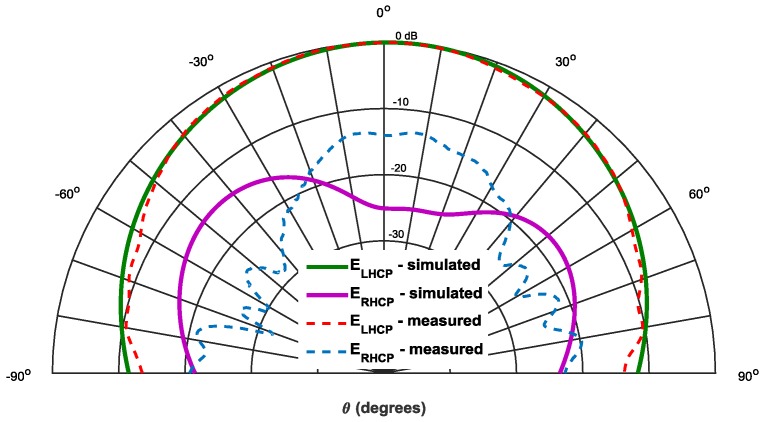
Simulated and measured radiation pattern of the designed circular patch antenna.

The Figure 4 shows a comparison between the simulated and measured radiation pattern of the antenna in terms of E_LHCP_ and E_RHCP_ components, for the radiation plane φ = 0°. A good agreement between simulation and measurements is visible. As expected, the patch has a wide radiation pattern, with a HPBW of 80°.

There is a complete relationship between the axial ratio (AR) and the E_LHCP_ and E_RHCP_ components.

(1) $$AR(dB)=20\log\left(\frac{\left|E_{LHCP}+E_{RHCP}\right|}{\left|E_{LHCP}-E_{RHCP}\right|}\right)$$

Considering the simulated and measured AR given from Figure 3 (shown for θ = 0°), respectively, *AR_meas_* = 3 dB and *AR_sim_* = 0.81 dB.

$$AR_{meas}=3\Leftrightarrow E_{LHCP}≅5.84\;E_{RHCP}\Leftrightarrow 20\log(E_{LHCP})≅\underset{\Delta_{1}=15.3\mathrm{dB}}{\underset{︸}{20\log(5.84)}}+20\log(E_{RHCP})\;AR_{sim}=0.81\Leftrightarrow E_{LHCP}≅21\;E_{RHCP}\Leftrightarrow 20\log(E_{LHCP})≅\underset{\Delta_{2}=26.4\mathrm{dB}}{\underset{︸}{20\log(21)}}+20\log(E_{RHCP})$$

These values of Δ_1_ and Δ_2_ are consistent with the rejection between simulated and measured components (shown in Figure 4), and the difference is justified by the slight deviation in the frequency of the axial ratio between simulated and measured values. However, a good quality of circular polarization is ensured.

### 2.2. Antenna Array Structure

Since the access lanes are typically large, the antenna for the RSU must have a wide radiation pattern in the horizontal plane, with a HPBW around 60°. With uniform antenna arrays, it is not possible to get an HPBW of 60° with a uniform gain across the lane width while ensuring low sidelobes, so non-uniform excitation techniques must be used.

The Fourier transform method (FTM) [9] was used in this array, since this method allows a feed distribution to be found for a set of array elements, so as to achieve a particular radiation pattern. For an odd number of elements (N = 2M + 1), each element feed coefficient can be calculated by the following:

(2) $$a_{m}=\frac{1}{T}\;\int_{-T/2}^{T/2}AF\left(\psi \right)\;e^{-jm\psi }\;d\psi \;=\frac{1}{2\pi }\int_{-\pi }^{\pi }AF\left(\psi \right)\;e^{-jm\psi }\;d\psi \;-M\leq m\leq M$$

where AF(ψ) represents the desired array factor and ψ *= kdcos* θ *+* β.

**Figure 5:**
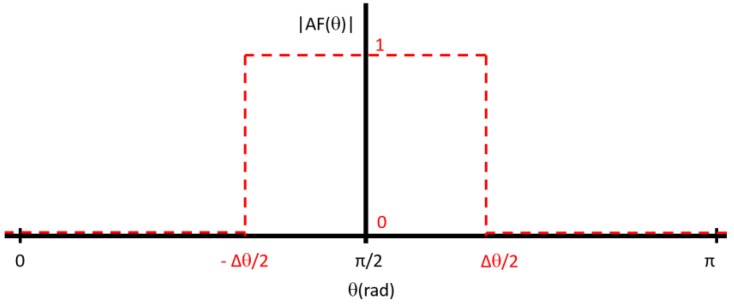
Desired array factor.

Figure 5 shows the desired array factor for this application, which consists of a uniform (maximum) amplitude over the lane width Δθ, and zero outside this zone. To provide some margin, since the HPBW is lower than the width between nulls of radiation pattern, the calculations were performed to Δθ = 70°. The integral of Equation (2) can be decomposed by

(3) $$a_{m}=\frac{1}{T}\;\int_{-\Delta \theta /2}^{\Delta \theta /2}1\;e^{-jm\psi }\;d\psi \;\Rightarrow a_{m}=\frac{1}{2\pi }{\left[-\frac{1}{jm}\;e^{-jm\psi }\right]}_{-\Delta \theta /2}^{\Delta \theta /2}\;-M\leq m\leq M$$

resulting in the following expression that allows the weights to be calculated for each *m*-element of this specific array.

(4) $$a_{m}=0.57\frac{\sin(0.57\pi m)}{0.57\pi m}\;-M\leq m\leq M$$

**Figure 6:**
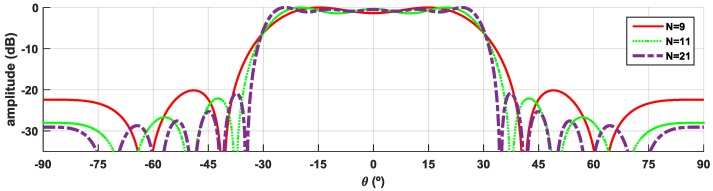
Variation of theoretical array response with the number of elements of the array, using Fourier transform method (FTM) coefficients.

Figure 6 shows the theoretical estimation of the array factor, using distance between elements of *d_1_* = 0.5λ, and with the corresponding weights calculated via FTM applied to each element by varying the number of elements N.

**Figure 7:**
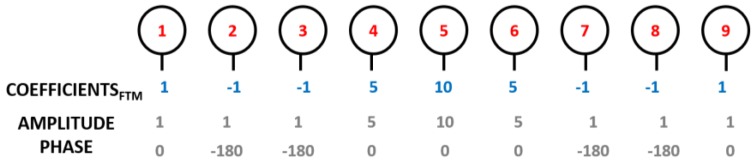
Antenna array weights.

According to Figure 6, it is possible to observe that by increasing N, the radiation pattern clearly becomes better defined, and with lower main lobe ripple; however, it dramatically increases the complexity of the array and the necessary AFN. The N = 9 elements was chosen, since it is the best trade-off between the desired radiation characteristics, the good performance, and the design complexity of the array. The array of feed distribution (weight to apply to each element) for N = 9 elements is presented in Figure 7.

Using a linear array of nine elements arranged along the horizontal plane (1 × 9), the radiation pattern in the vertical plane has a wide HPBW, not fulfilling the DSRC standard, which establishes a maximum of 70° in this plane. To meet that requirement, another line element was inserted, placed at *d_2_* = 0.6λ and with equal feed distribution, so that the final antenna array consists of a planar structure of 2 × 9 elements.

### 2.3. Array Feed Network

**Figure 8:**
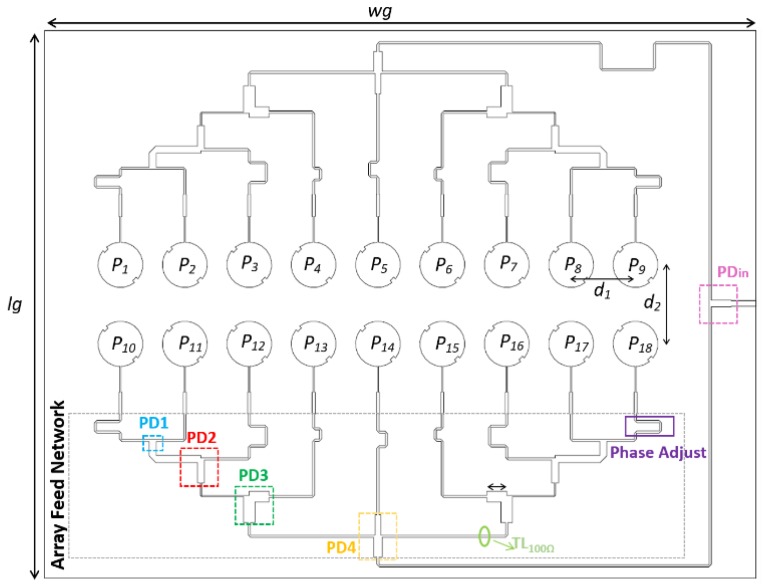
Antenna array structure.

The AFN has a great importance in the array design, implementing the feed distribution shown in Figure 7. In this case, it was designed to provide simplicity, a low profile, and ease of manufacture on a single dielectric layer structure placed in the plane of the radiating elements. Figure 8 shows the structure of designed array, with the AFN created using microstrip lines (with 100 Ω characteristic impedance), T-junction power dividers (PDs), and quarter-wavelength impedance transformers.

The AFN has a symmetrical arrangement with two branches (fed by PDin divider), compensating the physical rotation between the two lines of nine elements by a 180° phase delay between the two branches. The amplitude distribution was accomplished using unequal power dividers (PD1, PD2, PD3, and PD4) with different ratios. Additionally, each patch was fed by different length of lines, to individually adjust the feed of all the microstrip elements, P_1_... P_18_, with the desired phases.

**Figure 9:**
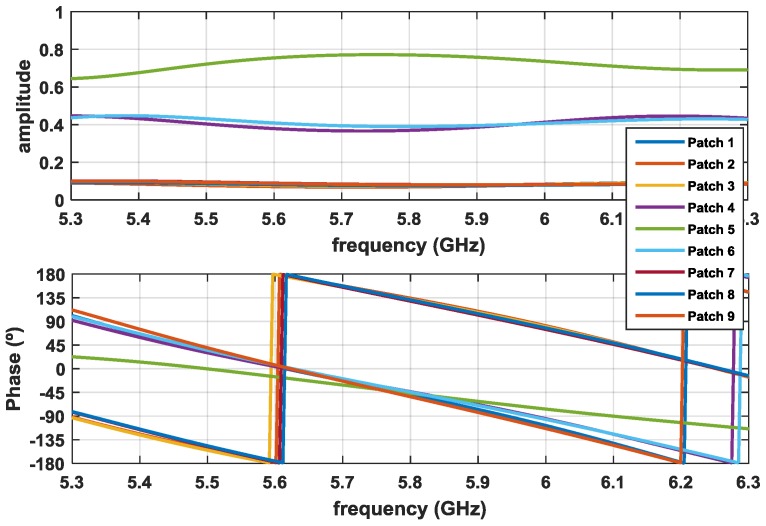
Amplitude and phase distribution of the array feed networks (AFNs).

The array was designed and simulated using the Computer Simulation Technology’s Microwave Studio (CST MWS) electromagnetic simulator [10]. Figure 9 shows the simulation results of the signals arriving at each patch of one branch (identical in the other branch) in terms of amplitude and phase. For the 5.8 GHz frequency, these values are summarized in Table 1. It is possible to conclude that both results—amplitude and phases—are consistent with the desired theoretical relationships expressed in Figure 7.

## 3. Results

**Figure 10:**
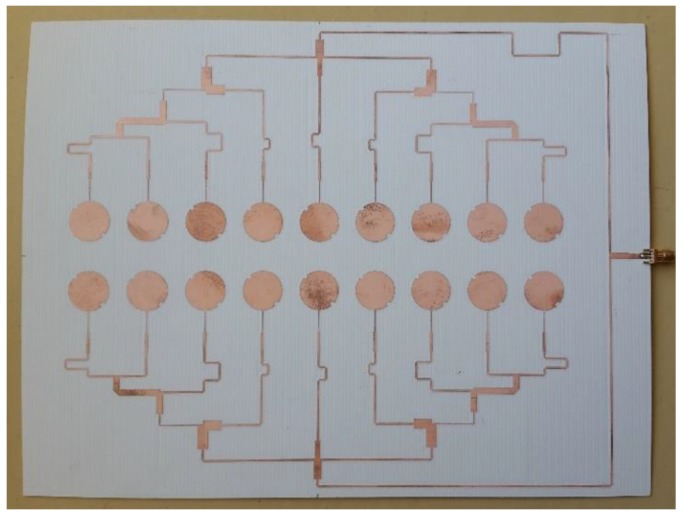
Photograph of the antenna array.

The antenna was designed, simulated, and manufactured using the Rogers RO4725JXR substrate, whose main characteristics are: dielectric constant *ε_r_* = 2.55, thickness *h* = 0.78 mm, and loss tangent *tg_δ_* = 0.0026. The prototype is shown in Figure 10, and presents global dimensions *W_g_ × Lg* = 286 × 218 mm^2^.

### 3.1. Reflection Coefficient

**Figure 11:**
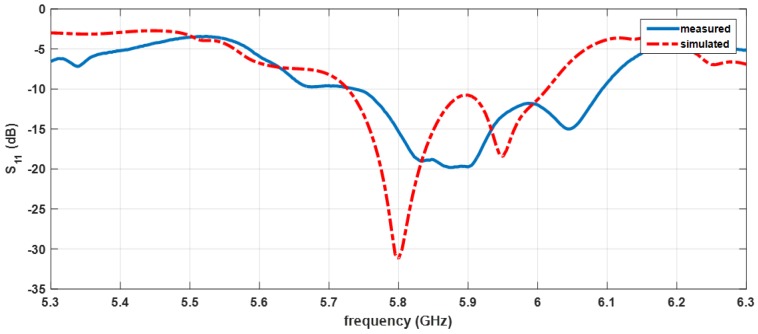
Simulated and measured reflection coefficient (S_11_) of the antenna.

The prototype was measured, and a comparison between the simulated and measured S_11_ of the antenna is presented in Figure 11.

The first impression that can be taken is a slight frequency deviation; however, it does not affect the array’s performance, showing a good agreement between simulated and measured results. Assuming as a common criterion for an impedance matching an S_11_ < −10 dB, the antenna has a bandwidth of 358 MHz.

### 3.2. Polarization

**Figure 12:**
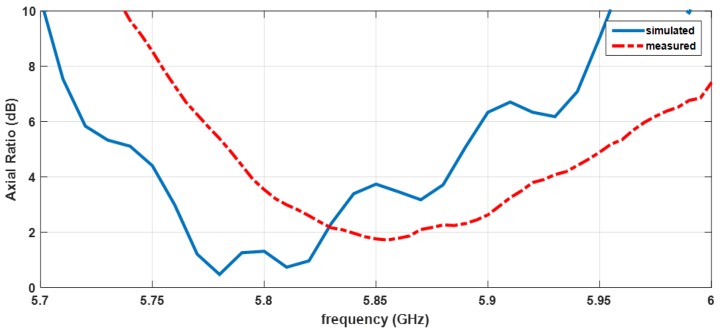
Simulated and measured axial ratio of the antenna with the frequency.

The Axial Ratio (AR) is a parameter that allows the quality of the circular polarization of an antenna to be characterized. Figure 12 illustrates the simulated and measured ARs of the designed antenna array. It is possible to observe an acceptable agreement between the two results, with only a small deviation.

Assuming an antenna with the AR < 3 dB as a well-accepted criterion for good circular polarization, it can be seen that the antenna array has a band around 100 MHz in which it presents a good measured AR.

### 3.3. Radiation Pattern

**Figure 13:**
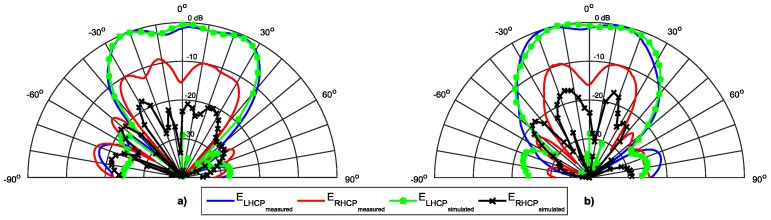
Normalized radiation pattern at 5.8 GHz. (**a**) Plane φ = 0°; (**b**) Plane φ = 90°.

**Figure 14:**
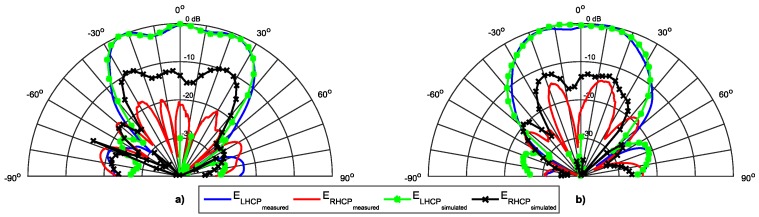
Normalized radiation pattern at 5.85 GHz. (**a**) Plane φ = 0°; (**b**) Plane φ = 90°.

**Figure 15:**
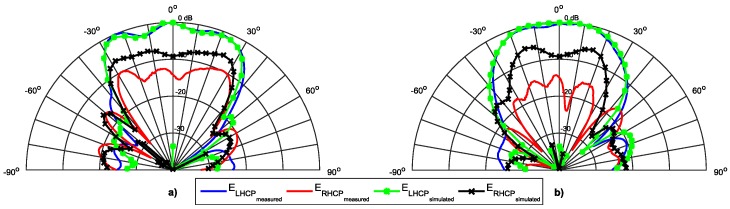
Normalized radiation pattern at 5.9 GHz. (**a**) Plane φ = 0°; (**b**) Plane φ = 90°.

Figure 13, Figure 14 and Figure 15 show the simulated and measured radiation pattern of the array prototype at three different frequencies in the two main radiation planes, φ = 0° and φ = 90°. In each figure, the left (E_LHCP_) and right (E_RHCP_) components of the electric field are presented.

For a straightforward analysis, the main radiation parameters are summarized in the Table 2. The first aspect that can be seen is the good agreement between the simulated and measured results, particularly in the dominant E_LHCP_ components.

The sidelobes are very low (close to −20 dB), while the HPBW in the horizontal plane is around 60° (as desired), with a magnitude that tends to be uniform, and in the vertical plane is near 50°, complying to the DSRC standard. The array presents a radiation efficiency close to 85% at 5.8 GHz, and a measured gain of 11.3 dBi.

The E_RHCP_ component has a small variation, and the rejection between the two components are consistent with the frequency shift in the AR graph. The better simulation results happen at 5.8 GHz, while due to the frequency shift in AR, the better measured values are at 5.85 GHz. Because of this shift in frequency of the AR, the measured ratio between the E_LHCP_ and E_RHCP_ is worse than the simulated at 5.8 GHz, and is better at 5.85 GHz.

**Figure 16:**
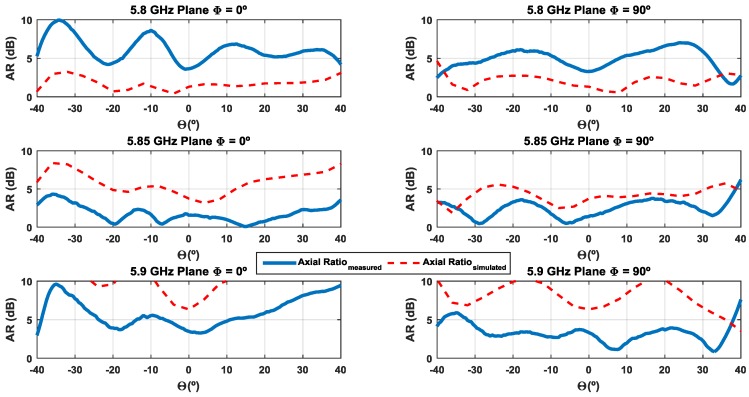
Simulated and measured axial ratio of the antenna over the mainlobe.

Figure 16 shows the axial ratio as a function of the main lobe angles of the radiation pattern at the three measured frequencies in the two main planes. The values are in agreement with the expected, taking into account the slight shift in the frequency of axial ratio and the rejection between components observed in the radiation pattern. Assuming a rejection greater than 15 dB and greater than 10 dB—corresponding to an axial ratio less than or equal to 3 dB and 5.7 dB, respectively Equation (1)—it is possible to observe that the DSRC standard is ensured at 5.85 GHz.

## 4. Discussion and Conclusions

Vehicular communications is a topic with great attention and development, and it is expected that in the future vehicles will interact with each other, and with their surrounding environment through road infrastructures, serving as sensors on the road, collecting and sharing data. DSRC is a technology used to implement this vehicular network with multiple applications (such as electronic payments) that has increasing use due to its convenience to all stakeholders.

In Europe, there are two main types of tolling; either the vehicles pass through toll plazas divided into several separated lanes, some using electronic tolling and other with manual payments, or they pass through free flow structures in which the payment is only electronic and the various lanes are not separated, allowing passage without reducing the speed. A reduced-speed passage is required in the first configuration, while the second requires a more complex system due to the presence of large interference between lanes.

A new approach consists of bringing the tolling to the access roads due to the simplicity that it allows the whole system; however, due to the wider dimensions of these lanes, the RSU module must have an antenna having a wider radiation pattern to cover the entire lane.

This work has allowed an antenna with an innovative radiation pattern to be developed—almost uniform throughout its mainlobe, suitable for the access roads of highways, and with negligible sidelobes, avoiding interference of vehicles circulating in peripheral areas. Furthermore, the simple structure of the array—designed in a single layer—allows for the integration of the feeding network together with the elements. Additionally, this antenna enables a good agreement between simulated and measured results, and a wide bandwidth covering the DSRC frequencies.

## Figures and Tables

**Table 1:** Array Feed Network–Simulated Amplitude and Phase (5.8 GHz).

Patch	Amplitude	Phase
1	0.073	−50°
2	0.07	134°
3	0.073	131°
4	0.37	−45°
5	0.77	−47°
6	0.39	−45°
7	0.08	128°
8	0.076	130°
9	0.08	−54°

**Table 2:** Radiation pattern characteristics. HPBW: half power beam width.

Frequency (GHz)	5.8		5.85		5.9	
Plane (φ)	0°	90°	0°	90°	0°	90°
HPBW_simulated_	62°	51°	62°	51°	60°	50°
HPBW_measured_	62°	51°	61°	49°	60°	48°
SLL_simulated_ (dB)	−21	−22	−22	−21	−19	−18.6
SLL_measured_ (dB)	−19	−21	−20.5	−21	−23	−21
